# Green Chemistry in Protected Horticulture: The Use of Peroxyacetic Acid as a Sustainable Strategy

**DOI:** 10.3390/ijms11051999

**Published:** 2010-05-03

**Authors:** Gilda Carrasco, Miguel Urrestarazu

**Affiliations:** 1 Departamento de Horticultura. Facultad de Ciencias Agrarias, Universidad de Talca, Chile; 2 Departamento de Producción Vegetal, Escuela Politécnica Superior, Universidad de Almería, Almería, 04120, Spain; E-Mail: mgavilan@ual.es

**Keywords:** green chemistry, phytotoxicity, horticulture, peracetic acid, postharvest life, food industry

## Abstract

Global reduction of chemical deposition into the environment is necessary. In protected horticulture, different strategies with biodegradable products are used to control pathogens. This review presents the available tools, especially for the management of protected horticultural species, including vegetables and ornamental plants. An analysis of the potential for degradable products that control pathogens and also encourage other productive factors, such as oxygen in the root system, is presented. Biosecurity in fertigation management of protected horticulture is conducted by using peroxyacetic acid mixtures that serve three basic principles: first, the manufacture of these products does not involve polluting processes; second, they have the same function as other chemicals, and third, after use and management there is no toxic residue left in the environment. The sustainability of protected horticulture depends on the development and introduction of technologies for implementation in the field.

## 1. Sustainable Protected Horticulture and Its Challenges

Production processes, especially those that are intensive [1,2], such as protected horticulture, degrade the environment to various extents. This one is the industry that in good part gives vegetables all over the world, due to the partial or total control of the conditions climatic of culture. The development of chemical products has permitted the food and protected horticulture industries to improve microbial safety [3]. However, the protected horticulture sector is expected to use low toxicity products that will maintain the sanitary control of vegetables grown in protected environments (greenhouses, tunnels and padding). Growers are required to combine good agricultural practices and standards of quality assurance, so food is healthy and attractive when consumed by the population [4]. This presents a global challenge for the protected horticulture industry.

One of the strategies recommended for the protected horticulture industry is the incorporation of new, environmentally friendly technologies, such as innovative green chemistry [5–7], as part of a policy-driven approach to prevent and reduce contamination. This strategy can be achieved by complying with the principles of green chemistry formulated by Anastas and Warner in 1998 (cited by [8]), which promote sustainability, efficiency and economy.

## 2. Principles of Green Chemistry

The principles of green chemistry are key tools for achieving sustainability, as they combine the possibility of synthesis of new products with conditions for assuring that they are degradable and non-toxic to the environment [5]. These principles are:

1. To prevent the generation of waste; prevention is better than treating waste after its formation.
2. To design synthesis methods to generate new products from start to finish.
3. To design chemical syntheses that are less toxic to human health and the environment.
4. To (re)design chemical products to preserve their efficacy but reduce toxicity.
5. To use safe solvents, separating agents and catalysts and not using these when possible.
6. To recognise the energy requirements for their environmental and economical impacts. The synthesis methods should take place at room temperature and pressure.
7. To use renewable materials.
8. To avoid chemical derivates. To use safe solvents and reaction conditions.
9. To use catalytic reagents (as selective as possible) in greater proportions than the stoichiometric reagents.
10. To design biodegradable products.
11. To analyse the chemical processes in real time to avoid contamination.
12. To minimise the risk of accident spills or other releases of chemicals.

## 3. Strategies of Green Chemistry in Protected Sustainable Horticulture: The Use of Peroxyacetic Mixtures

The use of sodium hypochlorite is questioned and not suggested as a green chemistry alternative for its application in agriculture for pathogen control or in fertilisers. Therefore, we consider the continual use of peroxyacetic acid mixtures in liquid solutions that meet all of the principles of green chemistry outlined above.

Peroxyacetic acid (C_2_H_4_O_3_), the reaction product of acetic acid (CH_3_COOH) and hydrogen peroxide (H_2_O_2_) in liquid solution, is a very strong oxidising agent, even more powerful than chlorine or chlorine dioxide. It is translucent and colourless and produces no foam, which makes it suitable for use in irrigation facilities. Peroxyacetic acid has a very pungent smell and a pH of 2.8, and must subsequently be stabilised [8]. Peroxyacetic acid mixtures are primarily used as sanitisers in the food industry [9], since they can control deposits, odours and biofilms on surfaces in contact with food, such as fresh fruits and vegetables [10]. The virucidal power of peroxyacetic acid has also been reported [11]. The chemical reactions related to acid peroxyacetic (or peracetic acid) are:

**Figure:**
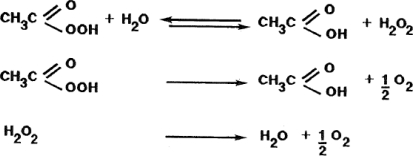

There are several registered products containing peracetic acid, hydrogen peroxide and acetic acid in different proportions as stabilised mixtures on the market. They can be used in the application of green chemistry in agriculture. None of the products formed during the degradation process (see above) are harmful. Acetic acid, hydrogen peroxide, water, and oxygen, are biodegradable, not harmful or even secondarily useful.

The specific mechanism of action of peroxyacetic acid mixtures for the control of biofilms, especially its ability to fix or remove it, is not well documented. The dissolved oxygen in a liquid medium has been described as a key factor that may be limiting for growth [12,13]. The optimum growth conditions are within a restricted range of dissolved oxygen, below which conditions are toxic and insufficient for growth.

The dose and technical management of peroxyacetic acid mixtures for the control of biofilms must be considered for efficient use and to prevent clogging of drippers or descaling. When applied in fertigation, three points should be considered and balanced for peroxyacetic acid mixture use:

1. To maintain control over pathogens when possible through its effect as a disinfectant [14];
2. The effects on secondary benefits, such as radical oxygenation on production [15]; and
3. The phytotoxic levels of each of its individual components.

Among the various functions of these peroxyacetic acid mixtures in protected horticulture and the food industry, several other uses include:

1. Disinfection of fertigation solution for control of pathogens [16];
2. Removal of drips through scaling [17,18];
3. Control of rainfall by preventing the formation of biofilms [16];
4. Increase of radical oxygenation due to the subproducts of decomposition, H_2_0, and O_2_, which have been evaluated in recirculation systems in soilless cultures [19];
5. Postharvest removal of natural microflora in freshly cut vegetables [10,19]; and
6. In other areas of the food industry, such as aquaculture, peroxyacetic acid mixtures control pathogens in fish by replacing other toxic products [20]. In molluscs, peroxides have also been evaluated as a promising alternative [21].

### 3.1. Use of Peroxyacetic Acid Mixtures for the Maintenance of Irrigation Facilities: Clogging, Environmental and Biosafety Control

Protected horticulture in south-eastern Spain constitutes a large proportion of greenhouses for the purpose of producing vegetables, and is characterized by the use of advanced irrigation and nutrition technologies. Some of the most important technological additions in this area and worldwide are localised irrigation and fertigation. The vast improvement of localised irrigation systems introduced in the past few decades has promoted more rational use of water resources, development and upgrading of marginal farming areas, as well as the use of low-quality water with high salt concentrations or even recycled water (treated water).

#### 3.1.1. Effects on Clogging

Drip irrigation consists of localised irrigation, which provides low flows (up to 16 L h^−1^ per emitter) [22], and are ideal for securing the soil or providing an adequate amount of water. This technique enables the establishment of a wet bulb to constitute a water reserve sufficient for the development of crop growth without significant losses by deep percolation [23]. Therefore, it is possible to achieve high efficiency in water use. In the holdings of western Almería, consumption of around 6,000 m^3^ ha^−1^ yr^−1^ can be found. By not wetting the entire soil, but only part of it, the plant concentrates its roots in the wet area so that it becomes necessary for nutrients to be taken up from the irrigated water, a practice known as “fertigation” [24]. Fertigation is commonly used in protected horticulture as it improves the benefits of drip irrigation and supports some new in relation to other disinfectants [23], maximises input, uses low-quality resources, and increases the production. This provides the agrosystem with a differential level of competitiveness and justifies the high initial cost of investment.

In places where the soil has been replaced by soilless cultivation techniques, in either the substrates only or by growing directly in a nutritious solution [24], the sustainability of the system relies on the incorporation of technologies that allow for recycling of the substrates. These substrates are present in the non-harvested plant material, which should ideally be recycled as compost [25,26]. However, to achieve this recycling management, the soil must be treated with biodegradable chemical products during fertigation and throughout the productive chain [27].

One drawback in the fertigation system is the blockage of water passage by particles, which compromises correct functioning during the time of installation [24]. The duration of the emitters must be maximised so that uniformity and efficiency of the applied water do not decrease over time; consequently, blockages, which have different origins: physical (suspended soils), chemical (formation of precipitates), biological (bacteria and algae) and their combinations, must be prevented and treated, especially in the case of buried drip irrigation systems, where visual inspection is more difficult. This concern is especially notable in the development of antimicrobial coatings, usually synthetic polymers, for internal irrigation pipes [27].

Paradoxically, clogging produces an intrinsic property, especially in the case of auto-regulation emitters. The irrigation is based in a water volume applied per unit of area and the reagent will be applied to a specific quantity of water for a given cycle. The plants that encounter a blocked emitter will not receive their volume of water, while the ones associated with a non-blocked emitter will receive more than the adequate amount of water (the emitters that function normally will be exposed to increased pressure). This phenomenon negatively influences the coefficient value of variation in the operation of the emitters [27].

The placement of several emitters per plant favours reduction of the clogging effects, because the deviation is lower. However, this greatly affects the uniformity; although two to eight emitters are installed per plant, there is an obstructed percentage of between 1 and 5% [28]. In addition, this measure is not economically viable.

The aspects mentioned above include the biosafety of the agrosystem, including water storage structures. In areas where water resources are scarce, the use of treated wastewater in urban sewage is common, and is increasingly used in protected horticulture. Although wastewater is of great interest, apart from other physical, chemical and physical-chemical problems [29], it may include a microbial level that represents a risk to human health. Under Spanish regulation, in the case of fecal coliforms, these are limited to 1,000 CFU (colony-forming units) per 100 mL for the use of irrigation water in vegetables that are to be consumed raw [30]. It is known that irrigation water is a vector of transmission of intestinal protozoa and nematodes that can cause diseases if ingested through crops irrigated with this water. In south-eastern Spain, there is contamination of irrigation water by several plant pathogens [31,32]. The presence of these pathogens, and in many cases their pathogenicity, has been demonstrated in irrigation waters [33]. Several authors, such as [34] and others, have demonstrated a high level of infectious agents, including some from the *Pythium sp*, *Phytophthora sp*, *Fusarium sp* and *Olpidium sp* families, in the irrigation waters of this region, even some reaching values above 80%. In the irrigation water, there may also be pathogenic organisms that are potentially harmful for humans and plants. These are introduced from the outside and can survive in irrigation water and adhere to biofilms to act as a reservoir, jeopardising the biosafety of the agrosystem. These biofilms are complex microorganisms and extracellular polymers fixed to a surface; they can be composed of a single or many species. Biofilms are available in any medium, provided that it is an environment with water and nutrients and that it can grow on hydrophobic or hydrophilic, and biotic or abiotic substrates [35]. This might indicate why organic matter is deposited at the interface of the water and surface, forming a layer that changes its physical properties, thereby improving the possibilities of adhesion of the bacteria. Thus, the problem of the clogging of the irrigation infrastructure by causes of biological origin is more obvious if the water used for irrigation is of residual origin. [27] classified the irrigation waters in terms of drip irrigation clogging, as indicated in Table 1.

Within the superficial uptake and use of residual wastewater, the frequent use of groundwater for irrigation is common. Most irrigation water used in arid and semiarid areas is characterised by a significant amount of dissolved bicarbonate. The natural terrains contain variable amounts of soluble salts and organic compounds, which are spread in the natural water supply. As a result, we assume that the global composition of underground water is remarkably constant, regardless of its geographic origin; the only change will be in the concentrations of each component. In regards to the amount of salt and the risk for clogging [27], established the following categories of irrigation water quality:

The dissolved ions normally precipitate by adding a precipitation germ to an initial nucleus (greater than 1 micron) to facilitate and accelerate the formation of saline precipitates that normally respond to microcrystallines. These initial geneses of the process are favoured by solid particles (under suspension) and promoted by the salinity of the fertigation water that contributes to waterlogging. The biofilm acts not only against rainfall due to its inherent biochemistry activity, but also constitutes a physical seat for the subsequent growth of precipitated salt layers [27].

#### 3.1.2. Effects on Environmental

This interaction between the direct chemical precipitation and the synergy with the biofilms plays an essential role for two reasons. First, its direct role on clogging has already been described as an important factor. However, it also provides a “refuge” for pathogens, and thus fails to produce an effect if it is protected [27].

Traditionally, treatment of irrigation water with an acidic liquid solution was thought to be the best option for maintaining the working conditions of the irrigation system [27]. However, other options should be considered in horticulture for sustainable management.

#### 3.1.3. Effects on Biosafety

Although the available evidence indicates that the use of chlorinated water (0.5–1.0 mg L^−1^) is safe for crops, the use of a sodium hypochlorite solution (which releases Cl_2_) has been extended in irrigation facilities to avoid clogging problems of emitters by biofilm formation. The biocidal activity of chlorine is dependent on the amount of hypochlorous acid present in the water and in contact with microbial cells. Although the mechanism of action is not entirely clear, it is predicted to inhibit enzymatic reactions and protein denaturation. These have a broad spectrum of activity (bactericidal, virucidal and sporicidal) and free chlorine retention has the most disinfectant activity [36]. The addition of sodium hypochlorite in water has the following chemical reaction:

$$\begin{array}{l} NaOCl+H_{2}O↔NaOH+HOCl\\ HOCl↔H^{+}+OCl^{-} \end{array}$$

The balance of this reaction sequence depends on several factors, such as the pH of water, which has an optimum range of 6.5 to 7.5 in order to be highly efficient and stable. In addition, the temperature and presence of organic matter affect its behaviour. The hypochlorous acid reacts with the organic matter present in the washing medium and, as a result of disinfection, sub-products such as trihalomethanes, chlorine fumes [34,37], chloroform (CHCl_3_) and bromodichloromethane (CHBrCl_2_) are formed. These products may be carcinogenic, mutagenic, teratogenic and/or toxic gases, which have been found to be directly related to the incidence of bladder cancer and birth defects [38–41].

### 3.2. Strategies for the Use of Peroxyacetic Acid Mixtures to Increase the Radical Oxygenation of Vegetables Growing in Soilless Cultures

The progressive deterioration of soil covered by structures in areas of horticultural production results from exhaustion, fungal contamination and salinisation. For these reasons, the incorporation of soilless culture techniques has been proposed as an alternative [42].

In this context, the use of hydroponic methods for obtaining safe products in less time and in a reduced surface has allowed for increased cultivation acreage. These include the use of special substrates for the production of fruits and vegetables and the recirculation of systems or of direct static nutrient solutions, used especially for cultivation of leafy vegetables [24].

Despite good results being obtained, there remains a need for greater efficiency in production systems. Research needs to be conducted on the aspects that may be limiting production, for instance, those caused by poor aeration in the rhizosphere that facilitate radical formation [14]. The improvement of radical oxygenation in these systems, called “floating systems” [43] or floating table systems, through peroxyacetic mixtures was investigated. There are several methods to increase oxygen availability in the rhizosphere, from the application of air pressure to the use of peroxyacetic mixtures in irrigation water (this study). These techniques also prevent aeration problems in sensitive crops, increase the efficiency of nutrient absorption and significantly increase productivity. However, we will only consider a limited scope of their use efficiency, as they can cause serious phytotoxicity problems that may even lead to the death of the plant [44]. In the case of arugula leaf vegetables (*Eruca sativa* Mill) consumed in salads, we evaluated different doses of a peroxyacetic acid mixture in the nutrient solution, where 40 mg L^−1^ of this mixture led to a better performance of the arugula when compared with plants cultivated in substrate (perlite) without the addition of this mixture [19].

### 3.3. Strategies for Using Peroxyacetic Acid Mixtures to Increase the Postharvest Life of Vegetables

The industry of producing horticultural products with minimum processing is based on the selection of the best raw materials, which are cultivated under strict production systems to conserve their oganoleptic and sanitary quality with minimised losses. Sustainable strategies are needed to maintain the final product quality through reduction of the microbial load in the production chain. However, the final washing step of the product will allow for disinfection. Therefore, the use of low doses of sodium hypochlorite is still necessary, although there may be reactions with the organic matter. For this reason, the food industry is searching for sustainable alternatives [45], such as peroxyacetic acid mixtures [10]. These researchers compared a peracetic acid mix and sodium hypochlorite as disinfectants on postharvest vegetables, the results indicated that the peracetic acid mixture was better for washing fruit and improving postharvest life, because it is better for the environment (due to low toxicity) and health safety, and does not affect the taste characteristics of the fruit.

### 3.4. Other Uses of Peroxyacetic Mixtures in the Food Industry

As mentioned previously, peracetic acid is a strong disinfectant with a broad spectrum of antimicrobial activity [45]. Both peracetic acid and hydrogen peroxide easily degrade into oxygen and water with the potential to replace formalin in the control of fish pathogens, such as the *Multifiliis ichthyophthirius* ectoparasite. The use of peroxyacetic acid mixtures is a possible alternative to be implemented in aquaculture, according to studies conducted by [20]. Other studies conducted on molluscs aimed to replace the chlorination and chemical agents used as descaling agents, which are toxic to marine species. For mussels, the first stages of life are the most vulnerable and therefore may require reduced doses of biocides. Therefore, commercial peracetic acid has proven to be an effective alternative for species such as the *Mytilopsis leucophaeata* and embryos of *Dreissena polymorpha*. However, future studies are needed to determine the optimal concentrations of these products [21].

## 4. Conclusions

It is possible to incorporate green chemistry strategies in the cultural practices of protected horticulture and the food industry and to release biodegradable elements that do not pollute the environment. It is necessary to investigate the adequate doses of different peroxyacetic acid mixtures that would be incorporated into soil nutrients, substrates, or even water systems for disinfection and improvement of radical oxygenation, which would positively affect crop yield. Moreover, during postharvest, it is important to improve the quality of healthy horticultural produce demanded by the population. There are many remaining questions to be answered. What is the life cycle of these biodegradable products? How will these processes reduce the carbon footprint of horticultural products obtained from different processes that use peroxyacetic mixtures? These questions will need to be answered through future investigations considering every crop and condition of the environment.

## Figures and Tables

**Table 1.** Effects of bacterial concentration on clogging risk [27].

**Clogging risk**	**Number of bacteria mL^−1^**
Low	<10,000
Medium	10,000–50,000
High	>50,000

**Table 2.** Effect of several parameters of irrigation water on clogging risk [27].

**Clogging factors**	**Clogging risk**		
	**Low**	**Medium**	**High**
pH	< 7.0	7.0–8.0	>8.0
Dissolved solids (mg L^−1^)	< 50	50–100	>100
Manganese (mg L^−1^)	< 0.1	0.1–1.5	>1.5
Total iron (mg L^−1^)	< 0.2	0.2–2.0	>2.0
Hydrogen sulphide (mg L^−1^)	< 0.2	0.2–2.0	>2.0
